# The roles of stomatal morphologies in transpiration and nutrient transportation between grasses and forbs in a temperate steppe

**DOI:** 10.1093/aob/mcad096

**Published:** 2023-07-20

**Authors:** Zhuo Chen, Hongbo Li, Wen-hao Zhang, Baolan Wang

**Affiliations:** State Key Laboratory of Vegetation and Environmental Change, Institute of Botany, the Chinese Academy of Sciences, Beijing 100093, China; College of Resources and Environment, University of Chinese Academy of Sciences, Beijing 100049, China; Institute of Environment and Sustainable Development in Agriculture, Chinese Academy of Agricultural Sciences, Beijing 100081, China; State Key Laboratory of Vegetation and Environmental Change, Institute of Botany, the Chinese Academy of Sciences, Beijing 100093, China; College of Resources and Environment, University of Chinese Academy of Sciences, Beijing 100049, China; State Key Laboratory of Vegetation and Environmental Change, Institute of Botany, the Chinese Academy of Sciences, Beijing 100093, China

**Keywords:** ecophysiology, functional groups, mineral nutrients, stomatal morphology, temperate steppe, transpiration, water-use efficiency

## Abstract

**Background and Aims:**

Grasses and forbs are dominant functional groups in temperate grasslands and display substantial differences in many biological traits, especially in root and stomatal morphologies, which are closely related to the use of water and nutrients. However, few studies have investigated the differences in nutrient accumulation and stomatal morphology-mediated transportation of water and nutrients from roots to shoots comparatively between the two functional groups.

**Methods:**

Here, we explored the patterns of accumulation of multiple nutrients (N, P, K, Ca, Mg and S) in leaves and roots, transpiration-related processes and interactions between nutrients and transpiration at functional group levels by experiments in a temperate steppe and collection of data from the literature.

**Key Results:**

The concentrations of all the examined nutrients were obviously higher in both leaves and roots of forbs than those in grasses, especially for leaf Ca and Mg concentrations. Grasses with dumbbell-shaped stomata displayed significantly lower transpiration and stomatal conductance than forbs with kidney-shaped stomata. In contrast, grasses showed much higher water-use efficiency (WUE) than forbs. The contrasting patterns of nutrient accumulation, transpiration and WUE between grasses and forbs were less sensitive to varied environments. Leaf N, P and S concentrations were not affected by transpiration. In contrast, leaf Mg concentrations were positively correlated with transpiration in forb species. Furthermore, linear regression and principal component analysis showed that leaf Ca and Mg concentrations were positively correlated with transpiration between the two functional groups.

**Conclusions:**

Our results revealed contrasting differences in acquisition of multiple nutrients and transpiration between grasses and forbs, and that stomatal morphologies are an important driver for the distinct WUE and translocation of Ca and Mg from roots to leaves between the two functional groups in temperate steppes. These findings will contribute to our understanding of the important roles of functional traits in driving water and nutrient cycling.

## INTRODUCTION

The temperate grassland in northern China is an integral part of the Eurasian grassland ecosystem with diverse ecological and agricultural functions (Kang *et al.*, 2007). Despite large variations in soil and vegetation types across the Eurasian steppe, the grassland ecosystem is composed mainly of perennial herbaceous species that are generally classified into two functional groups, namely grasses and forbs (Kang *et al.*, 2007; Schröder, 2009; Song *et al.*, 2012; Longo *et al.*, 2013; Tian *et al.*, 2021a; Zhou *et al.*, 2021). The two functional groups differ in many respects. For instance, there are distinct differences in root architecture and anatomical traits of absorptive roots between grasses and forbs in temperate steppes, and these differences drive variations in leaf physiological traits (Zhou *et al.*, 2021, 2022). Moreover, forbs and grasses in the steppe differ in their response to N enrichment, such that N addition enhances growth of grasses and suppresses growth of forbs (Song *et al.*, 2011; Tian *et al.*, 2021b). A nationwide investigation showed that forbs also exhibit higher foliar concentrations of mineral nutrients (e.g. N, P, K, Ca, Mg and S) than grasses (Han *et al.*, 2011).

Meta-analysis and experiments conducted across national or global terrestrial ecosystems along environment gradients indicate that in addition to N and P, other mineral nutrients (e.g. S, K, Ca and Mg) also play important roles in sustaining species diversity and stability of natural ecosystems (Han *et al.*, 2011; Fay *et al.*, 2015; Sandans and Peñuelas, 2015; Tian *et al.*, 2019, 2021; Xing *et al.*, 2021). Grassland productivity has been suggested to be limited by P and K in addition to N (Fay *et al.*, 2015). Therefore, exploring the patterns of these multiple nutrients in acquisition, transportation, accumulation and underlying mechanisms between grasses and forbs will help us to understand the potential effects of global changes on terrestrial nutrient cycling in grassland ecosystems (Han *et al.*, 2011; Tian *et al.*, 2019, 2021). However, most of our knowledge of mineral nutrients in the temperate steppe has come from studies on the function of N or P deposition in productivity or biodiversity, whereas limited information is available on mineral nutrient acquisition-associated characteristics and corresponding mechanisms.

**Fig. 1.**
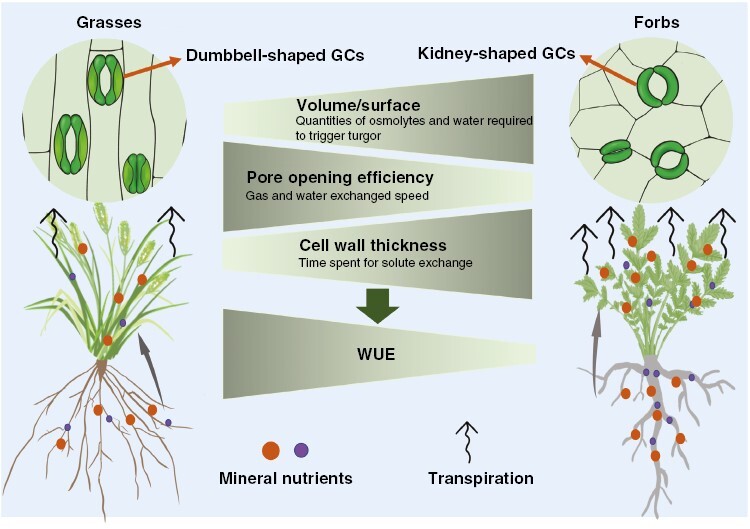
A proposal showing contrasting processes between grasses and forbs, including nutrient acquisition and accumulation, stomatal morphology-modulated transpiration and the involvement of transpiration in nutrient translocation from roots to leaves.

The accumulation of mineral nutrients in plants is determined by many factors, including nutrient availability, soil chemistry and plant trait-involved nutrient mobilization, uptake and transportation (Marschner, 2012; Lambers and Oliveira, 2019). In the same conditions, the abilities of uptake by roots and long-distance transportation via xylem are the primary drivers for nutrient acquisition and accumulation in roots and leaves, respectively (Marschner, 2012). It has been demonstrated that there are distinct differences in root architecture and anatomical traits of absorptive roots among grasses and forbs in temperate steppes (Zhou *et al.*, 2021, 2022). In contrast to grasses, forbs exhibit significantly higher stele diameter and vessel numbers (Zhou *et al.*, 2021). Given that these root traits are closely associated with water and nutrient uptake and long-distance transport (Steudle and Peterson, 1998; Rieger and Livin, 1999; Sperry *et al.*, 2002; Marschner, 2012; Wen *et al.*, 2019; Shen *et al.*, 2021; Wen *et al.*, 2022), it is assumed that these different root traits might lead to distinct nutrient acquisition and transportation to leaves between grasses and forbs (Fig. 1).

During the transportation of nutrients from roots to leaves via xylem, transpiration plays important roles (Masle *et al.*, 1992; Oliveira *et al.*, 2010; Marschner, 2012; Chen *et al.*, 2017). For instance, there is a close positive correlation between Ca distribution and transpiration rates (Tr) of shoot organs (Marschner, 2012). In leaves, >90 % of the total transpiration occurs through the stomata (Marschner, 2012). In contrast to the kidney-shaped guard cells (GCs) common to non-grass plants, grasses (Poaceae) form uniquely innovative stomata, which consist of two dumbbell-shaped GCs flanked by two lateral subsidiary cells (SCs) (Majore *et al.*, 2002; Hetherington and Woodward, 2003; Raissig *et al.*, 2017; Hepworth *et al.*, 2018; Nunes *et al.*, 2020; Fig. 1). The SCs help to support GCs mechanically and biochemically (Gray *et al.*, 2020). The stomata of grasses are developed in parallel rows within defined epidermal cell files, but the stomata are scattered throughout the epidermis in a less orderly manner than in non-grass plants (Chen *et al.*, 2017; Rudall *et al.*, 2017; Hepworth *et al.*, 2018). The dumbbell-shaped GCs enable the lower volume-to-surface ratio of the stomata of grasses, and this means that much smaller quantities of osmolytes and water are required to trigger cell turgor, which might lead to a lower energetic cost and lower transpiration (Hetherington and Woodward, 2003). In addition, this ‘graminoid’ morphology is associated with faster stomatal movements, leading to higher water-use efficiency (WUE) than kidney-shaped GCs (Johnsson *et al.*, 1976; Chen *et al.*, 2017; Hepworth *et al.*, 2018). Significant progresses have been made concerning stomata-regulated transpiration, but the studies are focused mainly on developmental biology or adaptation to stresses using individual plant species in the laboratory in controlled conditions. The characteristics of the two distinctly shaped stomata and their potential influence on physiology and ecological behaviours remain poorly understood in natural ecosystems.

The ecosystems of temperate grassland are dominated by grasses and forbs. Given the contrasting differences in root architecture, anatomical traits of absorptive roots and GC morphologies between grasses and forbs (Fig. 1), in addition to the close relationships between transpiration and mineral translocation to leaves via the xylem, we hypothesized that in temperate grassland ecosystem: (1) forbs would show significantly higher concentrations of mineral nutrients in both leaves and roots; (2) forbs with kidney-shaped stomata would have a significantly higher transpiration rate and lower WUE than grasses with dumbbell-shaped stomata; and (3) the varied transpiration elicited by distinct stomatal morphologies would be involved in contrasting long-distance transportation of nutrients from roots to leaves. In addition to experiments, more studies indicate that data collection or meta-analysis can provide strong evidence supporting scientific hypotheses (Han *et al.*, 2011; Song *et al.*, 2014; Tian *et al.*, 2019). In order to test our hypothesis, a combination of experiments and data collection from the literature was conducted in this study.

## MATERIALS AND METHODS

### Study sites

The plant samples were collected from a temperate steppe at the Duolun Restoration Ecology Station of the Institute of Botany, Chinese Academy of Sciences, in Duolun County, Inner Mongolia Autonomous Region, China (42°02ʹN, 116°17ʹE, 1324 m above sea level). This site belongs to the semi-arid continental monsoon climate zone. The mean annual temperature is 2.1 °C, and mean annual precipitation is 385.5 mm, with 80 % of total precipitation falling from June to September. The soil type is Calcisols, with 62.7 % sand, 20.3 % silt and 17.0 % clay. The mean soil bulk density is 1.31 g cm^−3^, and pH is ~7.0. The temperate steppe is dominated by several perennials, including the grasses *Stipa krylovii*, *Agropyron cristatum* and *Leymus chinensis* and the forbs *Potentilla bifurca*, *Artemisia frigida*, *Sibbaldia adpressa*, *Potentilla tanacetifolia*, *Potentilla acaulis* and *Phlomis pratensis* (Zhou *et al.*, 2021).

### Measurement of leaf photosynthetic rates, transpiration and WUE

A LI-6400 XT portable photosynthesis system equipped with an LED leaf cuvette (Li- Cor, Lincoln, NE, USA) was used to measure the leaf photosynthesis rate (Pn), transpiration rate (Tr) and stomatal conductance (Con) of the field perennials in the temperate steppe. The WUE was calculated as the ratio between Pn and Tr. The fully expanded leaves of each plant species were selected for measurement on sunny days. Leaf gas exchange was measured between 08:30 and 11:30 h in August 2021. Leaves were illuminated at 1500 mol m^−2^ s^−1^ using the LED light system. The photosynthetic rate, transpiration rate and stomatal conductance (the quantitative rate of how much water vapour or carbon dioxide passes through stomata) were recorded. Then the leaf area was measured by ImageJ (https://imagej.net/ij/index.html) after the leaf was photographed. For each species, four replicates were tested from different individuals, and the average value of each species was used for further analysis. Five grass species and 14 forb species were sampled in this study, and the detailed information of these plant species is provided in the Supplementary Data (Table S1).

### Plant sampling and measurement of element concentrations in leaves and roots

Individuals of selected plant species in the field were collected with a spade. The roots were shaken to remove loosely attached soil. About five individuals of each species were collected randomly for one replicate, and four replicates were collected for each species. After removal of the soil, shoots and roots were washed gently several times with distilled water. After washing, leaves and roots of grasses and forbs were subsampled. The whole roots and full expanded intact leaves were collected from all the individuals and mixed together for each species. Those leaves and roots were put into an oven at 105 °C for 30 min as quickly as possible and dried at 75 °C to constant weight. Then samples of leaf and root powder (~0.1 g) were put into Teflon tubes, 5 mL concentrated nitric acid was added, and they were left to soak overnight. Before digestion, 2 mL of hydrogen peroxide was added to the Teflon tubes. After that, shoots and roots were digested by microwave digestion (CEM MARS, Matthews, NC, USA). The digestion solution was transferred to a volumetric flask, and the solution was adjusted to a final volume of 50 mL with distilled water. The digested solutions were analysed by inductively coupled plasma emission spectrometry (ICP-AES) (iCAP6300, ICP-OES Spectrometer; Thermo Fisher, Waltham, MA, USA). Standard solutions were prepared by diluting stocks, and standard curves of P, S, K, Ca and Mg with correlation coefficients >0.99 were used. All measurements were run in triplicate for standards and samples, in order to ensure accuracy of the instrumental methods and analytical procedures. The concentrations of minerals (P, S, K, Ca and Mg) in 19 plant species were measured. Four and three replicates were tested in each grass species and each forb species, respectively, and the average value of each species was used for further analysis. The N concentrations in the leaves and roots were measured with an elemental analyser (Vario EL III, Elemental Inc., Hanau, Germany).

**Data collection of leaf nutrient concentrations**

We collected data from the published literature on leaf N, P, S, K and Ca concentrations from a temperate steppe located in Xilin River Basin in northern China (Chen and Wang, 2000). There were 20 grass and 72 forb species in the collected data (Supplementary Data Table S2).

**Measurement of specific leaf area**

For a replicate, ten fully expanded, healthy intact leaves of each species were collected randomly from about three individuals, immediately placed between two sheets of moist filter paper, and stored in self-sealing bags under refrigeration. Initially, the leaves were scanned (Expression 10000XL; Epson, Suwa, Japan), then the leaf area (LA) was measured using ImageJ software (https://imagej.nih.gov/ij/). The scanned leaves were oven dried at 65 °C to a constant mass. The average leaf area and dry weight of one leaf was calculated. The average value was regarded as a replicate, and ten similar replicates were sampled and recorded. Specific leaf area (SLA) was calculated as LA divided by leaf dry weight and is shown in the Supplementary Data (Table S3). The units of Tr and Con were converted from mmol H_2_O m^-2^ s^-1^ into mmol H_2_O g^-1^ s^-1^ according to the SLA during the correlation analysis between transpiration/conductance and nutrient concentrations.

### Data collection of leaf photosynthetic rates, transpiration and WUE

We collected data from the published literature on photosynthetic rate, transpiration rate and WUE in grasses and forbs across Chinese grassland ecosystems, and we found two studies with multiple grass and forb species (Supplementary Data Table S4; Ren *et al.*, 2015; Shen *et al.*, 2019). The sampling sites are an alpine swamp meadow in Gansu, China, and an alpine meadow in Qinghai, China, respectively.

### Statistical analysis

Initially, we divided the herbaceous species into two plant functional groups: grasses and forbs. Then an independent-sample *t*-test was conducted to detect the significance of nutrient concentrations and transpiration-related processes between the two functional groups. A linear regression was used to explore the relationships between nutrient concentrations and transpiration or stomatal conductance. These statistical analyses were carried out using the software SPSS (v.23.0).

## RESULTS

### Grasses and forbs differed in nutrient acquisition

**Fig. 2.**
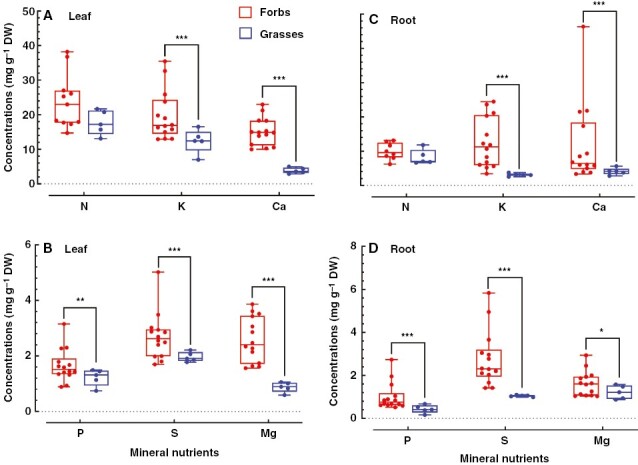
Nutrient concentrations in leaves and roots of grasses and forbs from a temperate steppe in Duolun County. There were five grass and 14 forb species. Four and three replicates were tested in each grass and forb species, respectively, and the average value of each species was used. Signiﬁcant differences between grasses and forbs in the same nutrient are presented as: ***P ≤ 0.001; **0.001 < *P* ≤ 0.01; *0.01 < *P* ≤ 0.05.

We collected 19 common plant species in a temperate steppe and determined N, P, S, K, Ca and Mg concentrations in their leaves and roots. We categorized the 19 species into the two functional groups of grasses and forbs. No significant differences in both leaf and root N concentrations between forbs and grasses were detected, although forbs showed a high trend for leaf and root N concentrations compared with grasses (Fig. 2A, C). In contrast, the forbs exhibited higher concentrations than grasses of P, S, K, Ca and Mg in both leaves and roots (Fig. 2). In particular, leaf Ca and Mg concentrations of forbs were approximately three and four times higher, respectively, than those in grasses (Fig. 2A, B). Root K and Ca concentrations of forbs were almost four and three times higher, respectively, than those in grasses (Fig. 2C).

**Fig. 3.**
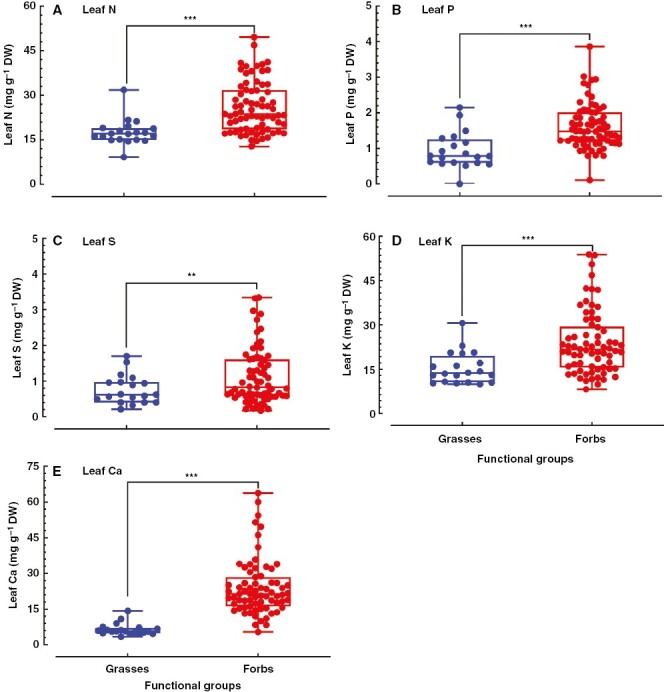
Nutrient concentrations in leaves of grasses and forbs from a temperate steppe in Xilin River basin. The data were collected from the published literature. There were 20 grass and 72 forb species in the collected data. Signiﬁcant differences between grasses and forbs are presented as: ***P ≤ 0.001; **0.001 < *P* ≤ 0.01; *0.01 < *P* ≤ 0.05.

To test whether this difference in mineral nutrients in forbs and grasses of the temperate steppe was a general pattern, we collected data about nutrient accumulation in the temperate steppe from published literatures. The plants were sampled in Xilin River basin in Inner Mongolia of northern China, and 20 grass and 72 forb species were included in the collected data (Chen and Wang, 2000). As shown in Fig. 3, all the examined leaf nutrients (N, P, S, K and Ca) were significantly higher in forbs than in grasses (Fig. 3A–E; Supplementary Data Table S2). These results suggest that higher concentrations of mineral nutrients in forbs than in grasses might be a common phenomenon in temperate steppes.

### Distinct transpiration rate and WUE between grasses and forbs

**Fig. 4.**
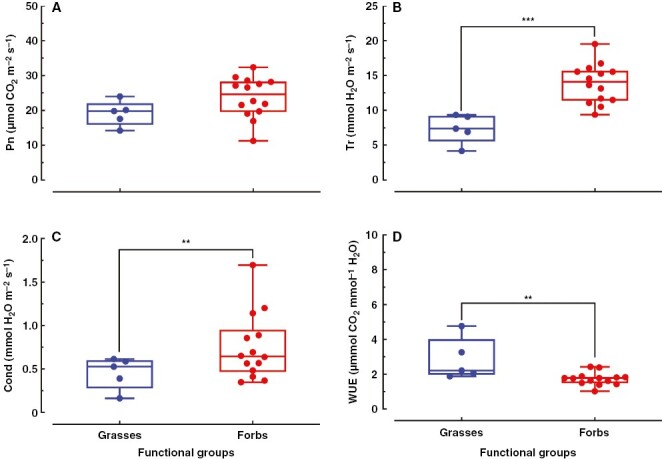
Net photosynthesis rate (Pn), transpiration rate (Tr), stomatal conductance (Cond), and water-use efficiency (WUE) of grasses and forbs. (A–D) There were five grass and 14 forb species in the temperate steppe. Significant differences between grasses and forbs are presented as: ***P ≤ 0.001; **0.001 < *P* ≤ 0.01; *0.01 < *P* ≤ 0.05.

Net photosynthetic rate (Pn) is a direct indicator of plant photosynthetic capacity. We found that there was no significant difference in photosynthetic rate between grasses and forbs in this temperate steppe (Fig. 4A). In contrast, the transpiration rate of forbs was obviously higher than that of grasses at both species and functional group levels (Fig. 4B; Supplementary Data Table S3). The stomatal conductance showed a similar tendency to the transpiration rate at both species and functional group levels (Fig. 4C; Supplementary Data Table S3). Contrary to transpiration rate and stomatal conductance, the WUE of forbs was markedly lower than that of grasses (Fig. 4D). In addition, data collection across grassland in China from an alpine swamp meadow in Gansu and an alpine meadow in Qing hai showed that there was no difference in photosynthetic rate between grasses and forbs, but the transpiration rate and WUE of forbs were significantly higher and lower, respectively, than those of grasses (Supplementary Data Fig. S1). These results indicate that the lower transpiration and higher WUE in grasses than in forbs is a common phenomenon in grassland ecosystems.

### Varied relationships between nutrient accumulations and transpiration between grasses and forbs

**Fig. 5.**
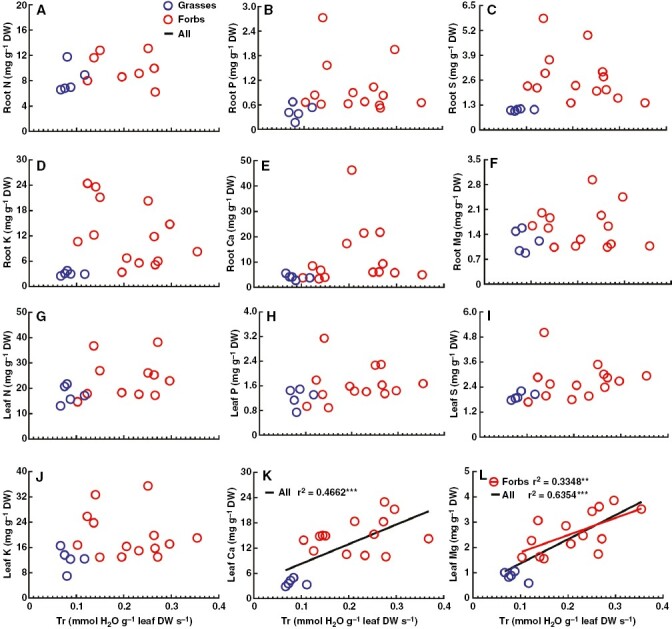
Linear regressions between root/leaf nutrient concentrations and transpiration rate at species (within the same group) or functional group levels in a temperate steppe. There were five grass and 14 forb species, and ‘All’ represents grasses + forbs. The significance of the linear regression and correlation coefficients is shown for each relationship. Signiﬁcant correlations are presented as: ***P ≤ 0.001; **0.001 < *P* ≤ 0.01; *0.01 < *P* ≤ 0.05.

In order to explore whether transpiration is responsible for the contrasting nutrient accumulation in roots and transportation to leaves between the two functional groups, linear regression analysis was conducted between nutrient concentrations in the root or leaf and transpiration rate, in addition to stomatal conductance, at both the species level within a functional group or at functional groups level (Fig. 5). For all the examined nutrients, there were no correlations between root nutrient concentrations and transpiration rates at both the species level within the same group and the functional group level (Fig. 5A–F). No correlations were found between transpiration rates and the three non-metallic nutrients (N, P and S) in leaves at both the species level within the same functional group and the functional group level (Fig. 5G–I). In contrast, a significant positive relationship between leaf Ca or Mg and Tr was found at the functional group level (Fig. 5K, L). Transpiration rate and stomatal conductance had no effect on leaf K, Ca and Mg at the species level within the grass group (Fig. 5J–L; Supplementary Data Fig. S2D). Stomatal conductance exhibited a significant negative correlation with leaf K, and the transpiration rate was positively correlated with leaf Mg concentration at the species level within the forb group (Fig. 5L).

**Fig. 6.**
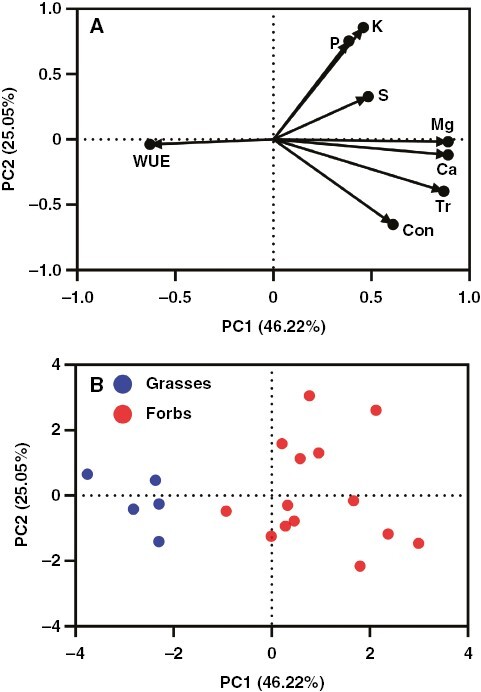
Principal component analysis for the leaf nutrients (with significant differences between the two functional groups), transpiration rate (Tr), stomatal conductance (Cond) and WUE from a temperate steppe. The figure shows nutrients, Tr, Cond and WUE loadings (A) and species distribution in trait place (B).

The results of principal component analysis were consistent with the linear regression analysis, which showed that of all the mineral elements, leaf Ca and Mg concentrations were most correlated with transpiration rate and stomatal conductance (Fig. 6A). In addition, the results revealed that the first two axes accounted for 46.22 % and 25.05 %, respectively (Fig. 6A). Correspondingly, none of the macronutrients was set in the same direction as the first axis with WUE (Fig. 6A). The species distribution in the graph of principal component analysis scores showed that the grasses separated from the forbs well and were located mainly on the first axis (Fig. 6B).

## DISCUSSION

In the present study, we examined the patterns of nutrient accumulation and stomatal morphologies modulated transpiration and explored the interactions of transpiration and nutrient accumulation between the functional groups of grasses and forbs in a temperate steppe. We found that both nutrient accumulation and transpiration-related processes exhibited contrasting functional group-dependent patterns, and stomatal morphologies are an important driver for the distinct WUE and translocation of Ca and Mg from roots to leaves between the two functional groups.

### Functional groups drive nutrient accumulations and transpiration

By analysing 11 elements in leaves of 1900 species across China, it has been demonstrated that most of the macronutrient concentrations in forb leaves are higher than those in grasses (Han *et al.*, 2011). This pattern of accumulation of nutrients is achieved across multiple climatic zones from the south to the north of China. Results from a local climate zone do not always follow the results obtained from large-scale investigation (Reich and Oleksyn, 2004; Han *et al.*, 2005; Townsend *et al.*, 2007; Luo *et al.*, 2015). Both our experimental results and the data collected from the literature showed that the examined macronutrient concentrations in forb leaves were significantly higher than those in grasses in the temperate steppe in northern China (Figs 2A, B and 3), indicating that accumulation patterns of mineral nutrients between grasses and forbs in temperate grasslands of northern China are in agreement with the nationwide patterns. In addition, studies from Britain also found that graminoids generally had lower macronutrient concentrations than non-grass herbaceous plants (Meerts, 1997, Güsewell, 2004). Given the consistency between the local and nationwide scale, we can conclude that the contrasting leaf nutrient accumulations between grasses and forbs are more conservative and are less sensitive to the environment at the functional group level.

In contrast to leaves, there is almost no information available for nutrient accumulation in roots of grasses and forbs. In this study, we found that, similar to leaves, most of the root macronutrient concentrations were markedly higher in forbs than in grasses (Fig. 2C, D), suggesting that forb species might be more efficient in mineral mobilization and uptake than grasses. Taken together, the significantly higher nutrients in both roots and leaves of forbs than in grasses imply that forbs are more efficient in nutrient uptake by roots and in transportation from roots to leaves.

In addition to accumulation of nutrients, our results and data collection from the literature also showed an obviously higher transpiration rate and stomatal conductance in forbs than in grasses (Fig. 4; Supplementary Data Fig. S1). In contrast to the kidney-shaped GCs common to most plants, grasses form uniquely innovative stomata, which consist of two dumbbell-shaped GCs flanked by two SCs (Hetherington and Woodward, 2003; Chen *et al.*, 2017; Hepworth *et al.*, 2018; Nunes *et al.*, 2020). The dumbbell-shaped GCs enable a lower volume-to-surface ratio of grass stomata, and this allows much smaller quantities of osmolytes and water to trigger cell turgor, which might lead to lower energetic cost and lower transpiration (Hetherington and Woodward, 2003). In addition, this dumbbell-shaped morphology is associated with faster stomatal movements, leading to higher WUE than kidney-shaped GCs (Grantz and Assmann, 1991; Chen *et al.*, 2017; Hepworth *et al.*, 2018; Nunes *et al.*, 2020). Grasses began to diversify in the late Cretaceous period and now dominate more than one-third of global land area (Chen *et al.*, 2017). The flexible stomatal movement helps grasses to adapt to changing environments, which gives grasses the evolutionary edge in their establishment across the globe (McKown and Bergmann, 2018). Based on the literature, we predicted that forbs should have a higher transpiration rate than grasses at both species and functional group levels in the temperate steppe of China. Our results for transpiration rate and stomatal conductance supported our ­prediction perfectly (Fig. 4; Supplementary Data Tables S3 and S4).

Water-use efficiency is the ratio of photosynthetic rate to transpiration rate (Hatfield and Dold, 2019). Given that there was less difference in photosynthetic rate between the two functional groups, grasses showed higher WUE owing to their lower transpiration rate than forbs (Fig. 4D). In addition, data collection from an alpine swamp meadow in Gansu and an alpine meadow in Qing hai across China showed similar results for photosynthetic rate, transpiration and WUE between grasses and forbs at species or functional group levels, respectively (Supplementary Data Fig. S1; Table S4), implying that the difference in transpiration between grasses and forbs is universal and independent of climate and topography in grassland ecosystems of China.

Based on these results, it is concluded that the distinct stomatal morphologies of grasses and forbs are an important mechanism underlying the contrasting transpiration and WUE at the functional group level.

### Transpiration is an important candidate driving contrasting nutrient accumulation between grasses and forbs

The long-distance translocation of water and mineral nutrients from roots to leaves is achieved mainly by xylem vessels (Jeschke and Hartung, 2000; Loepfe *et al.*, 2007; Marschner, 2012). The transpiration mass flow plays an important role in nutrient uptake (Russell and Barber, 1960; Mengel and Kirby, 1982; Shrestha *et al.*, 2021). Transpiration rate can affect nutrient uptake both directly and indirectly, through effects on the rate of radial transport of nutrients and by influencing the supply of nutrients to the plasma membrane of root cells (Lundegardh, 1955; Tinker and Nye, 2000; Cramer and Hawkins 2009; Houshmandfar *et al.*, 2018). Our results showed that the accumulation of all nutrients in roots was not affected by transpiration (Fig. 5A–F). The availability of nutrients in the soil is the most important factor determining their uptake by roots (Marschner, 2012). The absence of an interaction between root nutrients and transpiration might be caused by lower nutrient availability in the soils of the temperate grassland. In theory, the distribution of a nutrient element that is transported in the xylem should be related solely to the transpiration rate and the duration of transpiration (Marschner, 2012). It has been demonstrated that the total leaf mineral contents were positively correlated with transpiration efficiency in the plant *Sorghum* (Masle *et al.*, 1992). In fact, nutrient translocation via transpiration is dependent on many factors, such as the type of mineral, plant age, mineral availability, the proportion of xylem to phloem transfer, and so on (Oliveira *et al.*, 2010; Marschner, 2012). In otherwise comparable conditions (e.g. external concentration), the effects of transpiration rate on the transport of nutrients are variable, being strongly dependent on the plant species and type of nutrient (Marschner, 2012). Our results showed that leaf K and Mg concentrations were significantly correlated with stomatal conductance and transpiration rate within leaves of forb species, respectively, but none of them was affected by transpiration within the leaves of grass species (Fig. 5; Supplementary Data Fig. S2), indicating that plant species play large roles in the interactions between transpiration and the transportation of nutrients. In addition to plant species, the type of mineral is another important factor affecting the interactions between transpiration and the transportation of nutrients (Marschner, 2012). The interaction between leaf nutrient concentration and transpiration is usually absent or minor for N and P, but it might be significant for Ca or Mg (Shaner and Boyer, 1976; Engels and Marschner, 1993; Marschner, 2012). For instance, there is a close positive correlation between Ca distribution and the transpiration rates of shoot organs (Baas *et al.*, 2003; Marschner, 2012). In agreement with the literature, it was shown that concentrations of N, P and S in leaves were not affected by transpiration within both grass species and forb species (Fig. 5). In contrast, concentrations of Mg in leaves were positively correlated with transpiration rates within forb species (Fig. 5). More importantly, leaf Ca and Mg concentrations exhibited a significant positive correlation with transpiration at the functional group level (Fig. 5). The results of principal component analysis were consistent with the linear regression analysis, which showed that the concentrations of leaf Ca and leaf Mg were most correlated with the transpiration rate and stomatal conductance among all of the mineral elements (Fig. 6A, C). All these results indicate that transportation is an important driver for contrasting Ca and Mg distribution in leaves between grasses and forbs.

In addition to xylem, minerals can also be transported from roots to shoots by phloem, especially for more mobile nutrients (e.g. N, P, S and K; Hocking, 1980; Deeken *et al.*, 2002; Hafke *et al.*, 2007; Marschner, 2012). The absence of effects of transpiration rates on distribution of other minerals with a significant difference between grasses and forbs might be attributed to their higher proportion of xylem to phloem transfer in the stem tissue (Hocking, 1980; Jeschke and Pate, 1991; Marschner, 2012).

Absorption of minerals by the roots is the prerequisite for nutrient transportation. The rhizosphere processes, including root architecture, root exudation and mycorrhizal symbiosis, play important roles in acquisition of nutrients from the soil by plants (Shen *et al.*, 2021; Wen *et al.*, 2022). Given that many root traits (e.g. root architecture and root physiology) associated with rhizosphere effects are substantially different between grass and non-grass species (Zhou *et al.*, 2001; Wang *et al.*, 2022; Wen *et al.*, 2022), we speculate that rhizosphere processes might be involved in the contrasting accumulation of nutrients between grasses and forbs, and future studies aiming to elucidate functions of the rhizosphere in nutrient accumulation between the two functional groups are warranted.

### Conclusion

**Fig. 7.**
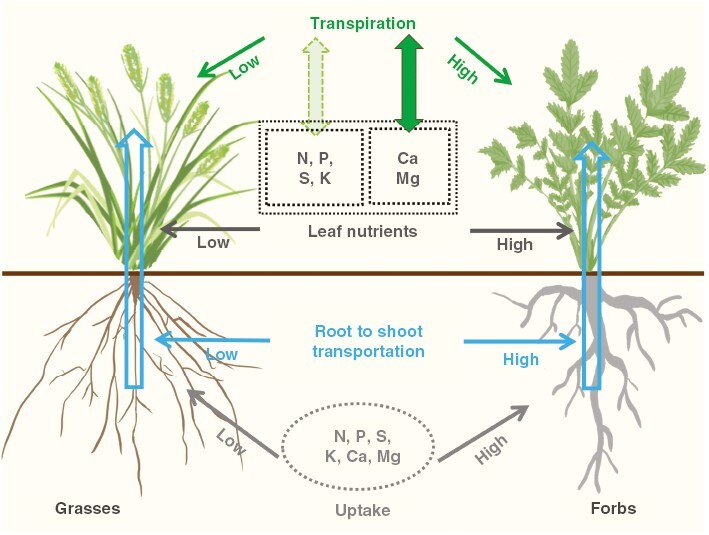
A summary illustrating different abilities between grasses and forbs in nutrient uptake, transportation, transpiration and interactions between leaf nutrient accumulation and transpiration.

In summary, we found that forbs were more efficient in the uptake, transportation and accumulation of macronutrients than grasses in temperate steppes; dumbbell-shaped stomata and kidney-shaped stomata are responsible for distinct transpiration and WUE, in addition to Ca and Mg transportation between grasses and forbs (Fig. 7). Given that all plants were sampled from the same environments in each experiment, in contrast to climate and soil factors, functional groups account for the largest variation for mineral accumulation and transpiration in temperate steppes, and stomatal morphologies are an important driver determining Ca and Mg flow and cycling in grassland ecosystems.

## SUPPLEMENTARY DATA

Supplementary data are available at *Annals of Botany* online and consist of the following.

Table S1: the list of grass and forb species sampled in this study. Table S2: concentrations of mineral nutrients in leaves of grass and forb species sampled from a temperate steppe in Xilin River basin. Table S3: net photosynthesis rate, transpiration rate, stomatal conductance, water-use efficiency and specific leaf area of the grass and forb species from a typical grassland in Duolun County. Table S4: net photosynthesis rate, transpiration rate and water-use efficiency of 46 plant species in Maqu alpine swamp meadow. Figure S1: photosynthetic rate, transpiration rate and water-use efficiency of grasses and forbs from published literature. Figure S2: linear regressions between leaf mineral nutrients and stomatal conductance at species or functional group levels in a temperate steppe.

mcad096_suppl_Supplementary_Material
