# Supplementary material for: The roles of stomatal morphologies in transpiration and nutrient transportation between grasses and forbs in a temperate steppe
Source: Ann Bot. 2023 Jul 20;132(2):229–39. doi: 10.1093/aob/mcad096 (PMC10583208; doi:10.1093/aob/mcad096)
Supplement: mcad096_suppl_Supplementary_Material [file mcad096_suppl_supplementary_material.docx]

**Table S1** The list of grass and forb species sampled in this study.

| **Species** | **Family** | **Cotyledon type** |
| --- | --- | --- |
| *Leymus chinensis* | Gramineae | Monocotyledon |
| *Stipa krylovii* | Gramineae | Monocotyledon |
| *Agropyron cristatum* | Gramineae | Monocotyledon |
| *Cleistogenes squarrosa* | Gramineae | Monocotyledon |
| *Koeleria macrantha* | Gramineae | Monocotyledon |
| *Allium tenuissimum* | Amaryllidaceae | Monocotyledon |
| *Allium senescens* | Amaryllidaceae | Monocotyledon |
| *Allium ramosum* | Amaryllidaceae | Monocotyledon |
| *Allium bidentatum* | Amaryllidaceae | Monocotyledon |
| *Astragalus leucocephalus* | Fabaceae | Dicotyledons |
| *Potentilla bifurca* | Rosaceae | Dicotyledons |
| *Potentilla tanacetifolia* | Rosaceae | Dicotyledons |
| *Potentilla acaulis* | Rosaceae | Dicotyledons |
| *Potentilla discolor* | Rosaceae | Dicotyledons |
| *Artemisia frigida* | Asteraceae | Dicotyledons |
| *Bupleurum chinenis* | Apiaceae | Dicotyledons |
| *Thalictrum petaloideum* | Ranunculaceae | Dicotyledons |
| *Aster altaicus* | Asteraceae | Dicotyledons |
| *Artemisia scoparia* | Asteraceae | Dicotyledons |

**Table S2** Concentrations of mineral nutrients in leaves of grass and forb species sampled from a temperate steppe in Xilin River basin. The data were collected from a published literature (Chen and Wang, 2000). There were 20 grasses and 72 forbs species respectively in the collected data.

| **Plan species** | **N**  (mg g^-1^) | **P**  (mg g^-1^) | **S**  (mg g^-1^) | **K**  (mg g^-1^) | **Ca**  (mg g^-1^) |
| --- | --- | --- | --- | --- | --- |
| **Grasses** |  |  |  |  |  |
| *Neotrinia splendens* | 17.5 | 1.33 | 0.4 | 13.49 | 7.22 |
| *Agropyron cristatum* | 15 | 0.62 | 0.96 | 9.92 | 6.82 |
| *Bromus inermis* | 21.2 |  | 0.89 | 22.9 | 5.56 |
| *Calamagrostis epigeios* | 18 | 1.17 | 1.53 | 12.94 | 9.01 |
| *Cleistogenes squarrosa* | 16.1 | 0.78 | 0.32 | 10.53 | 5.57 |
| *Elymus dahuricus* | 19.6 | 0.51 | 1.09 | 16.17 | 14.23 |
| *Festuca dahurica* | 9.2 | 0.57 | 0.56 | 10.31 | 3.39 |
| *Koeleria macrantha* | 14.7 | 0.76 | 0.49 | 15.89 | 4.6 |
| *Leymus chinensis* | 18.8 | 1.28 | 0.62 | 14.27 | 4.91 |
| *Psammochloa villosa* | 14.5 | 0.85 | 0.97 | 13.82 | 6.02 |
| *Puccinellia hauptiana* | 17 | 1.93 | 1.18 | 20.56 | 5.14 |
| *Stipa baicalensis* | 14.9 | 0.55 | 0.94 | 10.51 | 4.65 |
| *Stipa grandis* | 16.2 | 0.79 | 0.58 | 10.76 | 4.43 |
| *Stipa krylovii* | 16.4 | 0.92 | 0.63 | 10.31 | 5.7 |
| *Achnatherum sibiricum* | 21.7 | 0.6 | 0.4 | 12.99 | 6.17 |
| *Blysmus rufus* | 15.1 | 0.59 | 1.7 | 17.08 | 6.26 |
| *Carex duriuscula* | 19 | 1.07 | 0.54 | 20.29 | 5.69 |
| *Carex korshinskyi* | 17.4 | 1.49 | 0.21 | 20.64 | 10.85 |
| *Carex pediformis* | 17.1 | 0.74 | 0.4 | 13.09 | 5.11 |
| *Schoenoplectus tabernaemontani* | 31.8 | 2.15 |  | 30.62 | 7.5 |
| **Forbs** |  |  |  |  |  |
| *Koenigia divaricata* | 38.8 | 1.28 | 0.78 | 24.18 | 33.88 |
| *Rumex gmelinii* | 33.7 | 2.11 | 1.93 | 38.03 | 21.02 |
| *Urtica cannabina* | 34.6 | 1.68 | 1.71 | 21.2 | 63.79 |
| *Chenopodium acuminatum* | 49.6 | 2.92 | 3.32 |  | 46.12 |
| *Kali collinum* | 41.2 | 3.02 | 2.97 |  | 60.02 |
| *Suaeda glauca* | 24.4 | 2.83 |  | 26.58 | 20.09 |
| *Dianthus chinensis* | 18.5 | 1.48 | 0.78 | 25.41 | 28.44 |
| *Clematis hexapetala* | 22.1 | 0.106 | 1.68 | 13.23 | 21.65 |
| *Delphinium grandiflorum* | 25.4 | 2.03 | 2.46 | 42.33 | 49.61 |
| *Pulsatilla turczaninovii* | 15.8 | 1.04 | 0.23 | 15.06 | 25.99 |
| *Thalictrum squarrosum* | 28.6 | 1.14 | 1.11 | 21.49 | 22.36 |
| *Thalictrum aquilegiifolium* var. *sibiricum* | 31.8 | 1.13 | 2.03 | 19.74 | 24.14 |
| *Paeonia lactiflora* | 14.6 | 0.94 | 0.87 | 15.91 | 31.94 |
| *Descurainia sophia* | 46.9 | 2.54 |  | 53.58 | 51.51 |
| *Hylotelephium malacophyllum* | 27.6 | 2.17 | 0.6 | 20.95 |  |
| *Potentilla acaulis* | 17.5 | 1.64 | 0.35 | 13.55 | 13.14 |
| *Sibbaldianthe bifurca* | 23 | 2.79 | 0.98 | 15.25 | 17.64 |
| *Potentilla tanacetifolia* | 23.7 | 1.56 | 0.56 | 17.28 | 21.4 |
| *Sanguisorba officinalis* | 18.7 | 1.16 | 0.56 | 17.04 | 23.94 |
| *Spiraea aquilegiifolia* | 21.3 | 1.37 | 1.46 | 9.88 | 13.17 |
| *Spiraea pubescens* | 17.6 | 2.01 | 0.63 | 11.44 | 22.6 |
| *Astragalus galactites* | 38.2 | 1.3 | 1.1 | 15.67 | 23.88 |
| *Astragalus membranaceus* var. *mongholicus* | 40.4 | 1.86 | 0.63 | 22 | 23.51 |
| *Glycyrrhiza uralensis* | 39.9 | 1.82 | 1.27 | 26.07 | 20.69 |
| *Hedysarum gmelinii* | 34.2 | 1.36 | 2.88 | 14.44 | 33.95 |
| *Oxytropis filiformis* | 28 | 1.28 | 0.96 | 12.12 | 23.9 |
| *Oxytropis oxyphylla* | 27 | 1.09 | 0.69 | 11.84 | 21.97 |
| *Vicia sepium* | 33 | 1.65 | 0.48 | 14.94 | 15.07 |
| *Medicago ruthenica* | 39.8 | 1.88 | 0.17 | 16.01 | 17.98 |
| *Thermopsis lanceolata* | 40.9 | 1.22 | 1.23 | 18.54 | 18.56 |
| *Vicia unijuga* | 37.4 | 1.28 | 0.63 | 13.48 | 26.12 |
| *Polygala sibirica* | 22.4 | 1.41 | 0.68 | 8.2 | 8.34 |
| *Stellera chamaejasme* | 31.6 | 2.01 | 1.61 | 22.67 | 17.21 |
| *Chamerion angustifolium* | 20.9 | 1.73 | 0.62 | 11.61 | 11.97 |
| *Hippuris vulgaris* | 17.6 | 1.71 |  | 11.02 | 21.45 |
| *Pseudolysimachion dauricum* | 26.2 | 1.87 | 0.49 | 22.79 | 19.64 |
| *Pseudolysimachion incanum* | 16.8 | 1.71 | 0.84 | 19.76 | 14.29 |
| *Linaria vulgaris* subsp*. chinensis* | 22.4 | 1.46 | 0.58 | 27.89 | 18.71 |
| *Bupleurum scorzonerifolium* | 23 | 1.54 | 1.92 | 21.78 | 25.68 |
| *Saposhnikovia divaricata* | 17.2 | 1.76 | 1.63 | 20.29 | 5.41 |
| *Lappula myosotis* | 38.1 | 0.79 | 2.72 | 53.81 | 54.39 |
| *Leonurus sibiricus* | 22.8 | 2.3 | 1.64 | 23.68 | 25.07 |
| *Phlomoides mongolica* | 30.4 | 3.86 | 0.82 | 36.74 | 15.18 |
| *Schizonepeta tenuifolia* | 20.3 | 0.93 | 1.09 | 31.82 | 32.19 |
| *Scutellaria baicalensis* | 23.3 | 1.13 | 0.59 | 13.89 | 28.83 |
| *Scutellaria scordifolia* | 14.9 | 1.26 | 0.99 | 23.92 | 18.23 |
| *Thymus mongolicus* | 16.6 | 0.93 | 0.31 | 20.71 | 14.52 |
| *Cymbaria daurica* | 25.1 | 1.34 | 0.66 | 13.29 | 15.39 |
| *Pedicularis palustris* | 26.5 | 1.15 |  | 32.01 | 22.64 |
| *Pedicularis striata* | 18.4 | 1.48 | 0.68 | 22.54 | 32.58 |
| *Patrinia rupestris* | 17.2 | 0.93 | 0.79 | 20.85 | 15.75 |
| *Scabiosa comosa* | 17.4 | 1.49 | 1.74 | 19.59 | 17.32 |
| *Adenophora stenanthina* | 28.5 | 1.53 | 0.66 | 20.76 | 30.55 |
| *Filifolium sibiricum* | 17.1 | 0.99 | 1.19 | 23.98 | 20.31 |
| *Artemisia anethifolia* | 31.1 | 2.21 | 2.37 | 16.66 | 15.62 |
| *Artemisia frigida* | 27.3 | 2.24 | 0.5 | 23.08 | 9.93 |
| *Artemisia vestita* | 26.3 | 1.67 | 1.29 | 29.21 | 8.38 |
| *Aster tataricus* | 23.7 | 1.61 | 0.58 | 30.12 | 13.33 |
| *Cirsium esculentum* | 17.7 | 1.25 | 3.34 | 50.55 | 32.83 |
| *Aster altaicus* | 31.4 | 1.47 | 1.59 | 41.86 | 24.97 |
| *Ligularia mongolica* | 12.8 | 0.79 | 0.32 | 25.9 | 41.03 |
| *Saussurea alata* | 23 | 1.24 | 2.11 | 36.1 | 34.04 |
| *Olgaea lomonossowii* | 20.7 | 2.07 | 0.53 | 23.38 | 19.83 |
| *Allium tuberosum* | 38.5 | 2.94 | 1.36 | 46.83 | 18.64 |
| *Allium senescens* | 33.6 | 2.45 | 0.65 | 42.15 | 16.13 |
| *Anemarrhena asphodeloides* | 23.2 | 1.21 | 0.41 | 34.24 | 18.77 |
| *Asparagus dauricus* | 26.3 | 2.09 | 1.45 | 22.67 | 20.45 |
| *Hemerocallis minor* | 19 | 1.11 | 1.3 | 34.3 | 10.99 |
| *Veratrum nigrum* | 16.3 | 0.8 | 0.22 | 36.76 | 35.81 |
| *Belamcanda chinensis* | 20.1 | 1.26 | 0.32 | 20.38 | 25.94 |
| *Iris lactea* | 22.4 | 1.22 | 0.45 | 32.28 | 13.66 |
| *Iris tenuifolia* | 15.3 |  | 0.22 | 12.37 | 18.85 |

**Table S3** Net photosynthesis rate (Pn), transpiration rate (Tr), stomatal conductance (Cond), water use efficiency (WUE) and specific leaf area (SLA) of the grass and forb species from a typical grassland in Duolun County. The values represent means ± SE (n = 4 for Pn, Tr, Cond and WUE; n = 10 for SLA).

|  | **Pn**  (μmol CO_2_·  m ^–2^ ·s ^–1^) | **Tr**  (mmol H_2_O·  m ^–2^ ·s^–1^) | **WUE**  (μmolCO_2_·  mmol^–1^ H_2_O) | **Cond**  (mmol H_2_O·  m ^–2^ ·s^–1^) | **SLA**  (cm^2^ g^-1^ leaf DW) |
| --- | --- | --- | --- | --- | --- |
| **Grasses** |  |  |  |  |  |
| *Leymus chinensis* | 23.98 ± 1.08 | 7.37 ± 0.26 | 3.26 ± 0.13 | 0.61 ± 0.03 | 102 ± 3 |
| *Stipa krylovii* | 17.56 ± 1.14 | 9.33 ± 0.30 | 1.88 ± 0.07 | 0.59 ± 0.05 | 86 ± 2 |
| *Agropyron cristatum* | 20.11 ± 2.80 | 9.09 ± 1.08 | 2.22 ± 0.16 | 0.53 ± 0.07 | 129 ± 4 |
| *Cleistogenes squarrosa* | 19.81 ± 5.63 | 4.16 ± 0.39 | 4.62 ± 1.05 | 0.16 ± 0.02 | 211 ± 9 |
| *Koeleria macrantha* | 14.20 ± 1.56 | 6.90 ± 0.94 | 2.08 ± 0.07 | 0.39 ± 0.08 | 97 ± 3 |
| **Forbs** |  |  |  |  |  |
| *Allium tenuissimum* | 28.18 ± 0.57 | 15.55 ± 2.55 | 1.92 ± 0.23 | 0.64 ± 0.12 | 96 ± 1 |
| *Allium senescens* | 19.07 ± 2.43 | 11.70 ± 1.40 | 1.63 ± 0.07 | 0.37 ± 0.06 | 120 ± 3 |
| *Allium ramosum* | 22.68 ± 0.47 | 9.37 ± 0.78 | 2.46 ± 0.15 | 0.57 ± 0.05 | 146 ± 3 |
| *Allium bidentatum* | 21.55 ± 1.12 | 11.49 ± 0.26 | 1.87 ± 0.05 | 0.44 ± 0.06 | 96 ± 3 |
| *Astragalus leucocephalus* | 28.59 ± 3.19 | 16.06 ± 3.70 | 2.04 ± 0.45 | 1.70 ± 0.51 | 169 ± 1 |
| *Potentilla bifurca* | 21.85 ± 1.33 | 15.27 ± 2.10 | 1.48 ± 0.20 | 0.69 ± 0.14 | 128 ± 2 |
| *Potentilla tanacetifolia* | 27.12 ± 2.62 | 19.52 ± 2.18 | 1.42 ± 0.18 | 1.20 ± 0.19 | 136 ± 4 |
| *Potentilla acaulis* | 26.59 ± 3.25 | 14.77 ± 0.31 | 1.80 ± 0.22 | 0.89 ± 0.07 | 159 ± 3 |
| *Potentilla discolor* | 32.39 ± 3.60 | 13.63 ± 0.65 | 2.37 ± 0.21 | 0.65 ± 0.15 | 151 ± 4 |
| *Artemisia frigida* | 11.25 ± 1.44 | 11.06 ± 1.62 | 1.05 ± 0.15 | 1.14 ± 0.16 | 215 ±2 |
| *Bupleurum chinenis* | 29.55 ± 1.37 | 14.66 ± 3.72 | 2.13 ± 0.45 | 0.86 ± 0.16 | 177 ± 4 |
| *Thalictrum petaloideum* | 27.59 ± 3.86 | 15.54 ± 2.22 | 1.79 ± 0.14 | 0.48 ± 0.08 | 228 ± 11 |
| *Aster altaicus* | 19.71 ± 3.80 | 13.13 ± 1.30 | 1.58 ± 0.40 | 0.35 ± 0.05 | 191 ± 3 |
| Artemisia scoparia | 16.95 ± 4.37 | 10.51 ± 0.65 | 1.57 ± 0.31 | 1.20 ± 0.16 | 117 ± 3 |

**Table S4** Net photosynthesis rate (Pn), transpiration rate (Tr) and water use efficiency (WUE) of 46 plant species in Maqu alpine swamp meadow. The data were collected from a published literature (Ren et al., 2015; Shen et al., 2019). The values represent means ± SE (n = 5).

|  | **Pn**  (μmol CO_2_·  m ^–2^ ·s ^–1^) | **Tr**  (mmol H_2_O·  m ^–2^ ·s^–1^) | **WUE**  (μmolCO_2_·  mmol^–1^ H_2_O) |
| --- | --- | --- | --- |
| **Grasses** |  |  |  |
| *Agrostis perlaxa* | 5.03 ± 0.25 | 2.89 ± 0.11 | 1.77 ± 0.12 |
| *Agrostis hugoniana* | 8.43 ± 0.36 | 6.86 ± 1.02 | 1.39 ± 0.19 |
| *Festuca ovina* | 10.29 ± 0.51 | 6.61 ± 0.44 | 1.56 ± 0.03 |
| *Deschampsia caespitosa* | 11.05 ± 0.32 | 3.00 ± 0.18 | 3.76 ± 0.20 |
| *Koeleria macrantha* | 9.46 ± 1.25 | 4.68 ± 0.74 | 2.23 ± 0.16 |
| *Elymus nutans* | 5.85 ± 1.11 | 2.92 ± 0.67 | 2.20 ± 0.10 |
| *Agrostis hookeriana* | 6.63 ± 0.37 | 3.70 ± 0.40 | 1.90 ± 0.15 |
| *Poa pratensis* | 5.53 ± 0.61 | 3.52 ± 0.56 | 1.71 ± 0.16 |
| *Festuca sinensis* | 6.06 ± 0.35 | 3.39 ± 0.10 | 1.78 ± 0.05 |
| *Poa pachyantha* | 7.19 ± 0.57 | 6.26 ± 0.62 | 1.16 ± 0.03 |
| *Leymus secalinus* | 9.12 ± 0.61 | 5.66 ± 0.64 | 1.71 ± 0.10 |
| **Forbs** |  |  |  |
| *Hedysarum tanguticum* | 8.75 ± 0.06 | 6.39 ± 0.30 | 1.40 ± 0.07 |
| *Tibetia himalaica* | 10.65 ± 0.48 | 10.48 ± 0.53 | 1.07 ± 0.09 |
| *Oxytropis kansuensis* | 12.28 ± 0.56 | 8.77 ± 0.44 | 1.41 ± 0.03 |
| *Thermopsis lanceolata* | 13.95 ± 0.41 | 5.94 ± 0.54 | 2.50 ± 0.15 |
| *Ligularia virgaurea* | 13.40 ± 0.07 | 7.10 ± 0.64 | 1.99 ± 0.17 |
| *Cremanthodium pleurocaule* | 13.17 ± 0.52 | 5.81 ± 0.19 | 2.27 ± 0.06 |
| *Saussurea hieracioides* | 13.92 ± 0.22 | 10.67 ± 0.61 | 1.36 ± 0.05 |
| *Aster souliei* | 9.66 ± 0.38 | 11.38 ± 0.12 | 0.85 ± 0.04 |
| *Saussurea stella* | 8.08 ± 0.48 | 8.44 ± 0.58 | 0.96 ± 0.02 |
| *Cremanthodium lineare* | 13.88 ± 0.37 | 6.37 ± 0.23 | 2.21 ± 0.13 |
| *Anaphalis hancockii* | 5.96 ± 0.23 | 4.56 ± 0.26 | 1.34 ± 0.06 |
| *Leontopodium haplophylloides* | 10.61 ± 0.12 | 7.64 ± 0.63 | 1.45 ± 0.11 |
| *Anemone rivularis var. flore-minore* | 9.99 ± 0.50 | 6.54 ± 0.25 | 1.53 ± 0.07 |
| *Anemone coelestina var. linearis* | 10.73 ± 0.22 | 8.14 ± 0.24 | 1.32 ± 0.02 |
| *Trollius farreri* | 10.97 ± 0.80 | 7.96 ± 0.25 | 1.37 ± 0.06 |
| *Caltha palustris* | 8.44 ± 0.14 | 7.43 ± 0.20 | 1.15 ± 0.04 |
| *Thalictrum alpinum* | 13.03 ± 0.45 | 7.17 ± 0.18 | 1.81 ± 0.02 |
| *Rumex patientia* | 10.46 ± 0.23 | 6.73 ± 0.33 | 1.60 ± 0.09 |
| *Rumex aquaticus* | 7.14 ± 0.38 | 4.24 ± 0.30 | 1.77 ± 0.10 |
| *Polygonum sibiricum* | 19.23 ± 0.97 | 10.88 ± 0.11 | 1.77 ± 0.09 |
| *Polygonum viviparum* | 7.96 ± 0.32 | 7.83 ± 0.10 | 1.01 ± 0.03 |
| *Gentianopsis paludosa* | 12.21 ± 0.86 | 7.95 ± 0.86 | 1.60 ± 0.07 |
| *Halenia elliptica* | 9.18 ± 0.27 | 7.87 ± 0.11 | 1.17 ± 0.04 |
| *Gentiana leucomelaena* | 7.64 ± 0.72 | 4.95 ± 0.42 | 1.54 ± 0.03 |
| *Potentilla anserina* | 11.51 ± 0.21 | 8.48 ± 0.21 | 1.36 ± 0.01 |
| *Potentilla saundersiana* | 7.72 ± 0.31 | 7.50 ± 0.31 | 1.03 ± 0.01 |
| *Sanguisorba filiformis* | 13.31 ± 0.78 | 10.57 ± 0.77 | 1.27 ± 0.02 |
| *Allium sikkimense* | 6.04 ± 0.28 | 9.92 ± 0.26 | 0.62 ± 0.04 |
| *Allium chrysocephalum* | 15.71 ± 0.83 | 9.71 ± 0.37 | 1.63 ± 0.09 |
| *Veronica eriogyne* | 12.08 ± 0.65 | 8.90 ± 0.33 | 1.35 ± 0.04 |
| *Pedicularis rhinanthoides* | 4.25 ± 0.32 | 5.69 ± 0.61 | 0.77 ± 0.05 |
| *Euphorbia micractina* | 7.64 ± 0.18 | 8.32 ± 0.51 | 0.94 ± 0.04 |
| *Plantago depressa* | 7.09 ± 0.42 | 12.81 ± 0.29 | 0.56 ± 0.04 |
| *Nardostachys jatamansi* | 14.06 ± 0.93 | 11.75 ± 0.72 | 1.19 ± 0.01 |
| *Chamaesium paradoxum* | 7.02 ± 0.07 | 8.17 ± 0.05 | 0.86 ± 0.01 |


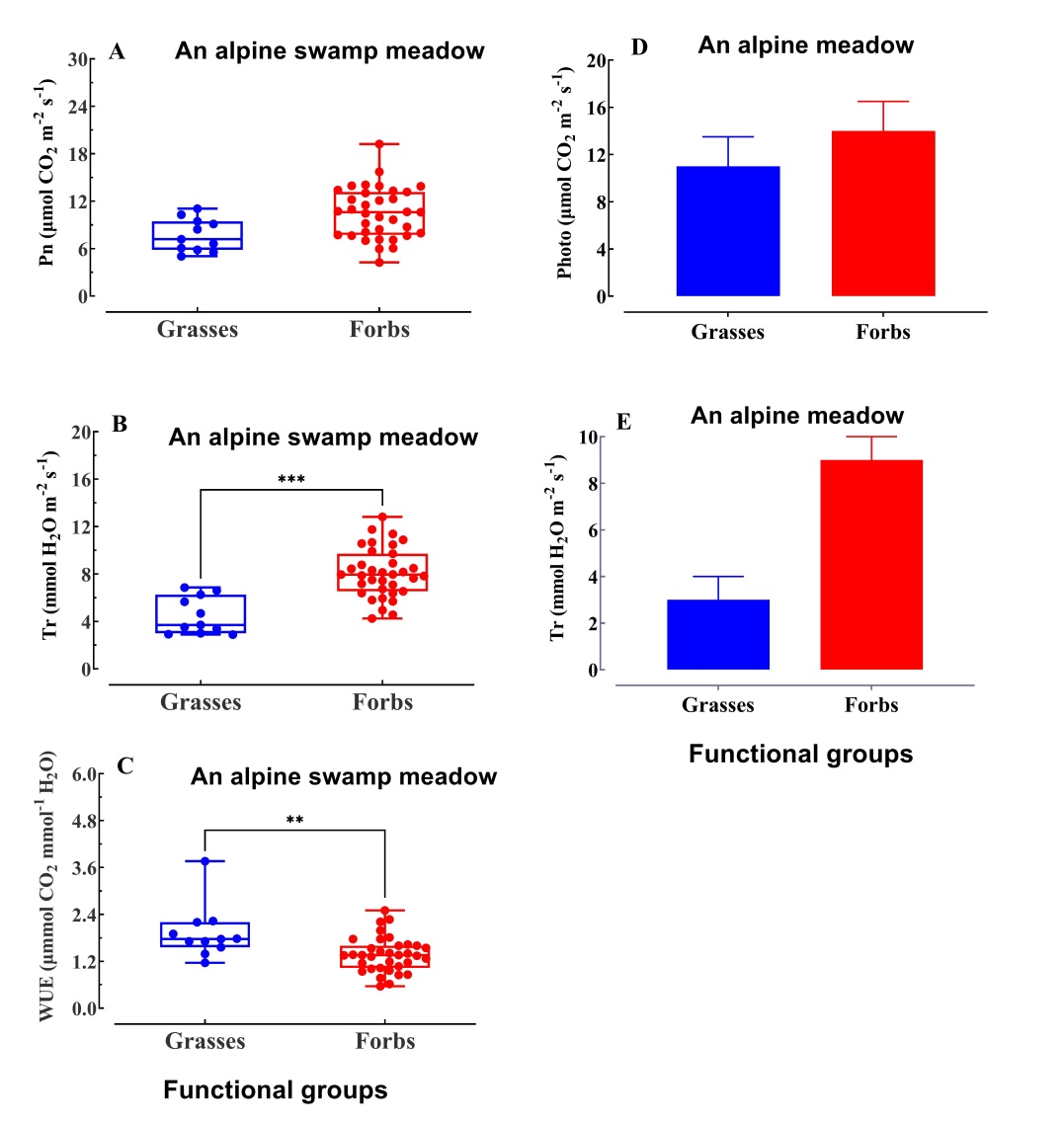


**Fig. S1** Photosynthetic rate, transpiration rate and WUE of grasses and forbs from published literatures. For the alpine swamp meadow, there were 11 grasses and 35 forbs species in the collected data. Five replicates were tested in each species, and the average value of each species was used (Ren *et al*., 2015). For the alpine meadow, 7 grasses and 12 forbs species were included (Shen *et al*., 2019).


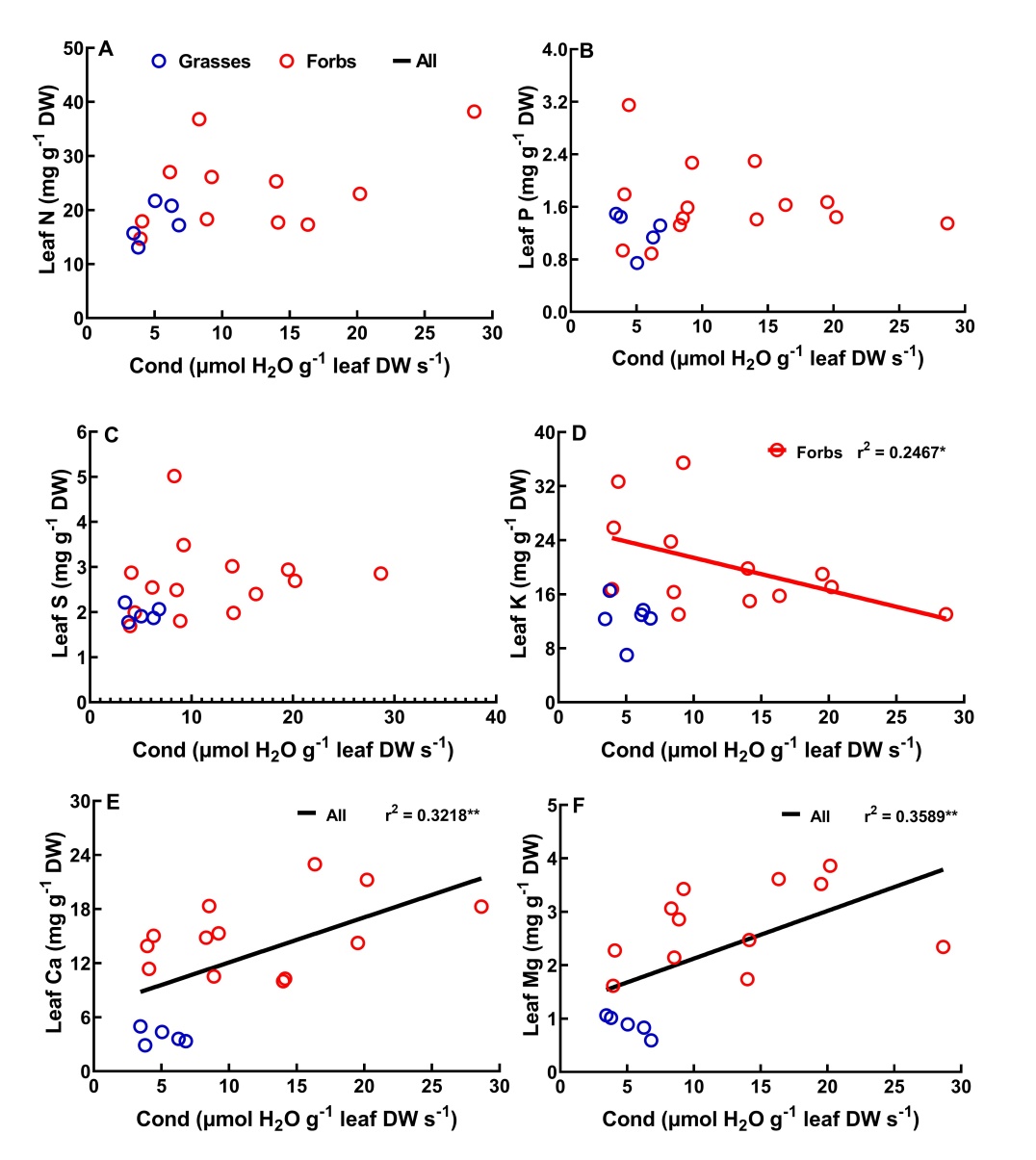


**Fig. S2** Linear regressions between leaf mineral nutrients and stomatal conductance at species (within the same group) or functional groups levels in a temperate steppe. There were 5 grasses and 14 forbs species respectively, and “All” represented grasses + forbs. The significance of the linear regression and correlation coefficients are shown for each relationship. Signiﬁcant correlations were presented with *** *P* ≤ 0.001; ** 0.001< *P* ≤ 0.01; * 0.01< *P* ≤ 0.05.
